# Mapping the O-GlcNAc Modified Proteome: Applications for Health and Disease

**DOI:** 10.3389/fmolb.2022.920727

**Published:** 2022-05-19

**Authors:** Rajan A. Burt, Ibtihal M. Alghusen, Sophiya John Ephrame, Maria T. Villar, Antonio Artigues, Chad Slawson

**Affiliations:** ^1^ University of Kansas Medical Center, Medical Scientist Training Program (MSTP), Kansas, KS, United States; ^2^ Department Biochemistry, University of Kansas Medical Center, Kansas, KS, United States

**Keywords:** O-GlcNAc, proteomics, mass spectrometry, PTM, OGT, OGA

## Abstract

O-GlcNAc is a pleotropic, enigmatic post-translational modification (PTM). This PTM modifies thousands of proteins differentially across tissue types and regulates diverse cellular signaling processes. O-GlcNAc is implicated in numerous diseases, and the advent of O-GlcNAc perturbation as a novel class of therapeutic underscores the importance of identifying and quantifying the O-GlcNAc modified proteome. Here, we review recent advances in mass spectrometry-based proteomics that will be critical in elucidating the role of this unique glycosylation system in health and disease.

**Clinical Trial Registration:** ClinicalTrials.gov, Identifier NCT05063539

## 1 Introduction

The addition of N-acetylglucosamine to serine and threonine residues of nuclear, cytoplasmic, and mitochondrial proteins (O-GlcNAc) is a poorly understood post-translational modification (PTM). The modification impacts thousands of distinct sites differentially across cell and tissue types (Wulff-Fuentes et al., 2021). Canonically, O-GlcNAc is regulated by two proteins: O-GlcNAc Transferase (OGT), which adds the modification to target substrates, and O-GlcNAcase (OGA), which removes the modification. OGT is conserved across metazoans (Kreppel et al., 1997; Lubas et al., 1997), and even observed in *Trichoplax adhaerens*, the simplest known animal (Selvan et al., 2015). Moreover, OGT is the most conserved glycosyltransferase encoded in the human genome (Jöud et al., 2018; Karczewski et al., 2020) The consequences of this unique glycosylation system are both pleotropic and enigmatic.

Although identified in the early 1980s, O-GlcNAc remains challenging to study (Torres and Hart, 1984). The regulation of O-GlcNAcylation by a sole pair of enzymes--and the necessity of each for mammalian life--makes it difficult to probe mechanistic questions. Deletion of either OGT or OGA is lethal in mice and rapidly growing cells (O'Donnell et al., 2004; Yang et al., 2012; Keembiyehetty, 2015). Pharmaceutical perturbations with OGA inhibitors result in a compensatory response where OGA is upregulated and OGT downregulated (Zhang et al., 2014). Both the neutral charge of an O-GlcNAc moiety and its mass addition do not provide a detectable change of mobility on standard polyacrylamide gel electrophoresis. Inducing constitutively active mutants at identified PTM sites is a powerful tool for phosphorylation events; however, the O-GlcNAc modification does not contain unique chemical moieties that can be mimicked with an amino acid substitution. In contrast, amino acid substitutions to permanently remove identified O-GlcNAc sites have been employed with success to explore cell signaling mechanisms (Housley et al., 2008; Myers et al., 2016; Wu et al., 2016; Kim et al., 2020) (reviewed in (Moon et al., 2022)).

O-GlcNAc is pleiotropic, influencing many biological processes. The efficiency of detained intron splicing inversely correlates with low O-GlcNAc levels while other splicing mechanisms remain unchanged (Tan et al., 2020). Pharmaceutical inhibition of OGA alters mitochondrial morphology and membrane potential (Tan et al., 2017), where OGA genetic knockouts in immortalized mouse embryonic fibroblasts alters electron transport chain composition and mitochondrial fission (St. Amand et al., 2018; Akinbiyi et al., 2021). OGT inhibits the 26S proteasome, with O-GlcNAc modifications identified via immunoblotting on 26S subunits (Zhang et al., 2003). These examples are inexhaustive, as the biological processes that OGT, OGA, and O-GlcNAc are implicated in are numerous. Moreover, aberrant O-GlcNAcylation reportedly characterizes several disease states, such as neurodegeneration (Ryan et al., 2019; Lee B. E. et al., 2020; Park et al., 2021), cancer (Shi et al., 2010; Ferrer et al., 2014; Sekine et al., 2018; Akella et al., 2020), and diabetes (Lehman et al., 2005; Masaki et al., 2020), among others (Fülöp et al., 2008; Umapathi et al., 2021). Mutations in the tetratricopeptide repeats catalytic domain of OGT is linked to human X-linked intellectual disability (Willems et al., 2017), and a single nucleotide polymorphism of OGA is a risk factor for type 2 diabetes mellitus (Lehman et al., 2005).

As evident, strategies that perturb OGT and/or OGA are instrumental when probing general phenotypic questions; however, the pleiotropic nature of this system makes mechanistic questions difficult to decipher with genetic knockouts or small molecule inhibitors. As our understanding of this system further develops, methods to map the specific sites of O-GlcNAcylation will be critical to elucidate mechanistic understanding. Techniques that can be applied to *in vivo* and patient-derived samples will be particularly important to investigate the role of this system in health and disease. This may become especially apparent with the advent of OGA inhibitors as a novel class of therapeutic (ClinicalTrials.gov Identifier: NCT04392271, ClinicalTrials.gov Identifier: NCT05063539).

### 1.2 Challenges in O-GlcNAc Proteomics

Mass spectrometric techniques to map the O-GlcNAc modified proteome and how glycosylation patterns change under different conditions could be powerful platform to assess the impact of O-GlcNAcylation. These strategies provide insight on the specific cellular signaling mechanisms regulated by OGT/OGA. Moreover, mapping specific O-GlcNAc sites provides candidates downstream of OGT/OGA that may be more amenable to perturbation and/or mutation. Typically, mass spectrometry-based proteomics requires high performance liquid chromatography coupled to tandem mass spectrometry (LC-MS/MS) to identify and quantify peptides and proteins (reviewed in (Aebersold and Mann, 2003)). Various LC-MS/MS data acquisition methods exist, but the most standard form is data-dependent acquisition (DDA), also referred to as “bottom-up” proteomics. In a brief and inexhaustive explanation, proteins are digested to peptides with protease(s) that follow specific patterns for enzymatic cleavage, most typically trypsin. Tandem mass spectrometry induces unimolecular dissociation of precursor ions (i.e., peptides) and generates associated mass-to-charge spectra of product ions (reviewed in (Aebersold and Mann, 2003)). Spectra are submitted to search engines to match them to their most likely peptide (a peptide-spectral match) (Mann and Wilm, 1994) [reviewed in (Eng et al., 2011)]. Peptide identities are deduced by these peptide-spectral matches, which then infers the identity of proteins present in the sample. As peptides elute from the coupled reversed-phase column to the electrospray ionization source of the instrument, the mass spectrometer selects the most intense ions for MS2 sequencing. This is the data-dependent acquisition component, as the mass spectrometer is making “on-the-fly” decisions to determine which ions present in the first data acquisition event (the precursor ions) to fragment and analyze in secondary data acquisition event.

Mapping PTMs using DDA methods may require enrichment of the modified peptides or proteins, depending on the stoichiometric abundance of the modification [reviewed in (Maynard and Chalkley, 2021)]. Unmodified peptides are more abundant than their modified counterparts, where the latter will be overshadowed in a complex mixture (i.e., a cell or tissue digested protein lysate). Enriching O-GlcNAc modified peptides poses unique challenges. The sub-stoichiometric amount O-GlcNAc modified peptides in a complex mixture necessitates strong enrichment sensitivity, particularly so for experiments with limited sample material. Glyco-protein modifications are diverse, where O-GlcNAc is one of many sugar modifications that could be enriched. N-acetylgalactosamine, an epimer of GlcNAc, also modifies serines and threonines (O-GalNAc) but canonically impacts extracellular and membrane-bound proteins (Rottger et al., 1998; Steentoft et al., 2011; Steentoft et al., 2013). The GlcNAc moiety itself may be incorporated into branched oligosaccharides. Traditionally, O-GlcNAc specificity is validated by checking the cellular localization of the putative modified protein. “True” O-GlcNAc hits are cytosolic, mitochondrial, and nuclear proteins, where O-GlcNAc modified proteins without these localizations are considered false positives. Although reasonable (as O-GalNAc and branched glycan modifications canonically occur via endoplasmic reticulum related pathways) this approach--by definition--limits the detection of O-GlcNAc modified proteins beyond the interior of the cell. This problem may be underscored as our understanding of O-GlcNAcylation evolves. For example, an alternative glycosyltransferase, EGF domain-specific O-linked N-acetylglucosamine transferase (EOGT), localizes itself to the endoplasmic reticulum and modifies secretory/membrane-bound proteins with O-GlcNAc (Matsuura et al., 2008; Sakaidani et al., 2011). Consequently, strategies for the unbiased discovery of O-GlcNAc modified proteins require robust enrichment specificity.

LC-MS/MS peptide sequencing techniques fragment precursor ions before the secondary data acquisition event. These product ions and the mass differences between them are used to deduce the peptide identity. For discovery DDA proteomics, the most typical fragmentation techniques employed are collisional-induced dissociation (CID) and higher-energy collisional dissociation (HCD). CID and HCD preferentially break the weakest bonds of the precursor ion. GlcNAc is connected to serine/threonine residues via a labile glycosidic bond (Huddleston et al., 1993; Chalkley and Burlingame, 2001; Jebanathirajah et al., 2003; Zhao et al., 2011). Subsequently, these collisional fragmentation techniques result in the near complete loss of the O-GlcNAc modification from the peptide backbone (Chalkley and Burlingame, 2003; Myers et al., 2013). This impairs the localization of the modification for peptides with multiple serine and/or threonine residues. Electron transfer dissociation (ETD) is an alternative fragmentation technique that specifically fragments the peptide backbone, preserving PTMs (Syka et al., 2004). ETD is a slower fragmentation process than collisional methods (Yu et al., 2017a). This can result in fewer peptide identifications. Precursor ion charge density impacts ETD fragmentation performance (Good et al., 2007; Liu and McLuckey, 2012; Frey et al., 2013). Precursors with higher charge density improve ETD fragmentation (Good et al., 2007; Liu and McLuckey, 2012; Frey et al., 2013). Non-covalent interactions can hold fragment ions together. This process reduces spectral quality and is otherwise known as non-dissociative electron transfer dissociation (Pitteri et al., 2005), (reviewed in (Riley and Coon, 2018)). As evident, the choice of data acquisition parameters for the identification and quantification of O-GlcNAc modified peptides is not necessarily straightforward.

### 1.3 Advances in Mapping the O-GlcNAc Modified Proteome

As previously noted, mapping the O-GlcNAc modified proteome poses a somewhat unique challenge in traditional bottom-up DDA proteomics, not only due to challenges with site localization when using collisional activation MS2 fragmentation techniques but also to limitations in sample enrichment. O-GlcNAc enrichment has a repertoire of diverse strategies, each with their own advantages and limitations. O-GlcNAc metabolic labeling techniques demonstrate success in numerous iterations, providing a remarkable platform to investigate diverse questions, (Boyce et al., 2011; Qin et al., 2017; Pedowitz et al., 2020) but cannot be readily applied to samples generated outside of laboratory tissue culture. Lectin weak affinity chromatography adopts widespread use as an inexpensive method for O-GlcNAc enrichment of native peptides, and has generated some of the largest datasets to date of the O-GlcNAc modified proteome (Trinidad et al., 2012; Maynard et al., 2020; White et al., 2020) but is limited by sample input requirements when working with scarce samples (Trinidad et al., 2012; Trinidad et al., 2013). Anti-O-GlcNAc antibodies show promise as a sensitive and specific method for native peptides with the capability to map over 1,000 unique sites with as little as 4 mg of input material (Burt et al., 2021) but are prohibitively expensive for some investigations. Antibody based methods may have limitations due to antigen-binding site specificity; however, polyclonal antibody mixtures have demonstrated significant coverage for multiple PTM spaces (Svinkina et al., 2015; Udeshi et al., 2020; Burt et al., 2021). Various O-GlcNAc antibody reagents exist, but to our knowledge, a comparison between these antibodies in their capability to map the O-GlcNAc modified proteome has not been formally investigated. For a more complete review of enrichment techniques for O-GlcNAc modified peptides, readers should refer to other reviews (Ma and Hart, 2014; Maynard and Chalkley, 2021; Yin et al., 2021; Zhu and Yi, 2021).

The labile glycosidic bond between a GlcNAc moiety and a serine/threonine residue necessitates innovative mass spectrometry data acquisition techniques. ETD was a key technological advancement for O-GlcNAc site specific mapping (Chalkley et al., 2009; Wang et al., 2010; Myers et al., 2013; Nagel et al., 2013). Some fragmentation strategies apply supplemental energy during or subsequent to ETD to overcome poor fragmentation from non-dissociative ETD events (Swaney et al., 2007; Ledvina et al., 2009; Frese et al., 2012). Activation of fragment ions post ETD fragmentation with high-energy collisional dissociation (EThcD) shows promise for O-linked glycopeptides (Yu et al., 2017b; Cao et al., 2020; White et al., 2020). HCD-product-triggered-ETD (HCD-pdt-ETD) fragments precursor ions with HCD, where upon detection of designated peak(s) in the MS2 scan, the mass spectrometer recollects another packet of ions of the same precursor for ETD or ETD with supplemental energy (Zhao et al., 2011). This strategy attempts to combine the advantages of both fragmentation methods and has shown promise for O-GlcNAc modified peptides (Zhao et al., 2011; Malaker et al., 2017; Riley et al., 2020; Burt et al., 2021). The primary HCD “scouting” scan serves as a filter for the subsequent ETD “localization” scan, only triggering the slower ETD scans upon detection of fragment peaks indicative of a HexNAc modification (Burt et al., 2021). This could be particularly useful for strategies with lower enrichment efficiencies, as ETD scan time is only designated for suspected O-GlcNAcylated precursors. Putative O-GlcNAcylated peptides have both HCD and ETD spectra for sequencing, enhancing confidence in the results (Lu et al., 2020).

**FIGURE 1:**
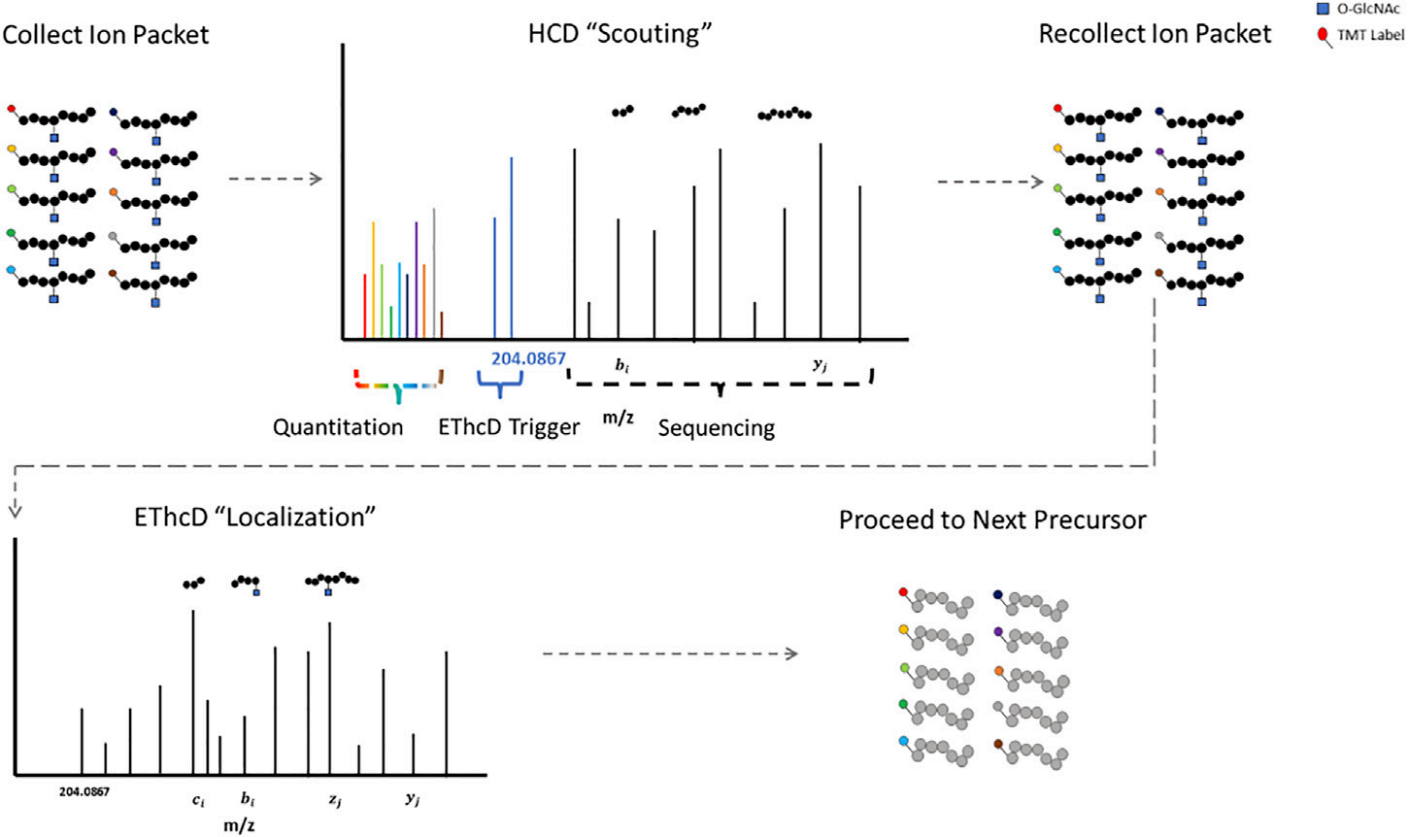
HCD-pdt-ETD for HexNAc Modified Peptides HCD-pdt-ETD methods couple collisional activation and electron transfer dissociation methods for putative O-GlcNAc modified precursors. Precursors are first fragmented with HCD, where upon detection of a HexNAc fragment ion (such as the 204.0867), a secondary packet of ions of the same precursor is recollected and fragmented with ETD. ETD provides an alternative fragmentation method that may preserve the HexNAc modification, allowing for site localization. ETD fragmentation can proceed with whatever preferred supplemental energy technique applied. The HCD “scouting” scan may serve as a filter to only trigger ETD on highly suspect O-GlcNAcylated peptides. In future investigations, TMT quantitation may be foreseeably coupled to the HCD “scout” scan.

Proteomic strategies to elucidate the biological functions of O-GlcNAc signaling will require both accurate site localization and robust quantification. Numerous strategies exist that allow for the quantitative analysis of multiple samples simultaneously, including but not limited to: isobaric mass tags (TMT, iTRAQ, DiLeu) (Thompson et al., 2003; Ross et al., 2004; Xiang et al., 2010; Chick et al., 2016; Mertins et al., 2018; Thompson et al., 2019; Li et al., 2020), SILAC (Ong et al., 2002), stable-isotope dimethyl labeling (Hsu et al., 2003), and hybrid isotopic mass difference and isobaric tag labeling strategies (Dephoure and Gygi, 2012; Evans and Robinson, 2013; Frost et al., 2018). Isobaric mass tag labeling with TMT is a popular method for quantitative proteomics. TMT relies on the quantification of reporter ions subsequent to fragmentation and was originally optimized for collisional activation fragmentation methods (Thompson et al., 2003; Li et al., 2020). HCD-pdt-ETD could circumvent this incongruity by providing TMT reporter ion quantification information via the “scouting” scan. Figure 1 illustrates how HCD-pdt-ETD strategies could provide TMT quantitative capability with ETD site localization. This strategy would also preserve the benefit of the HCD “scouting” scan serving as a filter for highly suspect O-GlcNAcylated peptides, improving coverage (Burt et al., 2021). Although not applying HCD-pdt-ETD methods, some investigators employ a parallel fragmentation approach where HCD spectra are collected for TMT reporter quantitation and EThcD spectra collected for peptide sequencing and O-GlcNAc site localization (White et al., 2020).

As mentioned previously, enrichment specificity is important when identifying putative O-GlcNAc sites. N-acetylglucosamine has stereoisomers. N-acetylgalactoseamine is one such stereoisomer that can also modify serine and threonine residues (O-GalNAc). This makes it challenging to distinguish these entities from their mass addition to fragment peaks alone. Consequently, putative O-GlcNAc sites are reported as HexNAc sites, highlighting stereoisomer ambiguity. O-GalNAc typically forms the basis of branched O-Glycans (reviewed in (Vasconcelos-dos-Santos et al., 2015)), but its singular addition may occur, such as the case with the Tn antigen (Ju et al., 2011; Cornelissen et al., 2020). O-GlcNAc and O-GalNAc modifications may have different biological implications, so the ability to distinguish between them would be useful. A singular addition of GlcNAc to asparagine residues may also occur (N-GlcNAc), further complicating specificity; however, N-GlcNAc appears to follow a consensus motif of N-X-S/T, where X can be any amino acid except proline, allowing for differentiation between the two modifications (Gavel and Heijne, 1990; Chalkley et al., 2009; Wang et al., 2010; Kim et al., 2012). Regardless, interpretation of putative O-GlcNAc modified peptides should be judicious as to not conflate these two distinct modifications.

**FIGURE 2:**
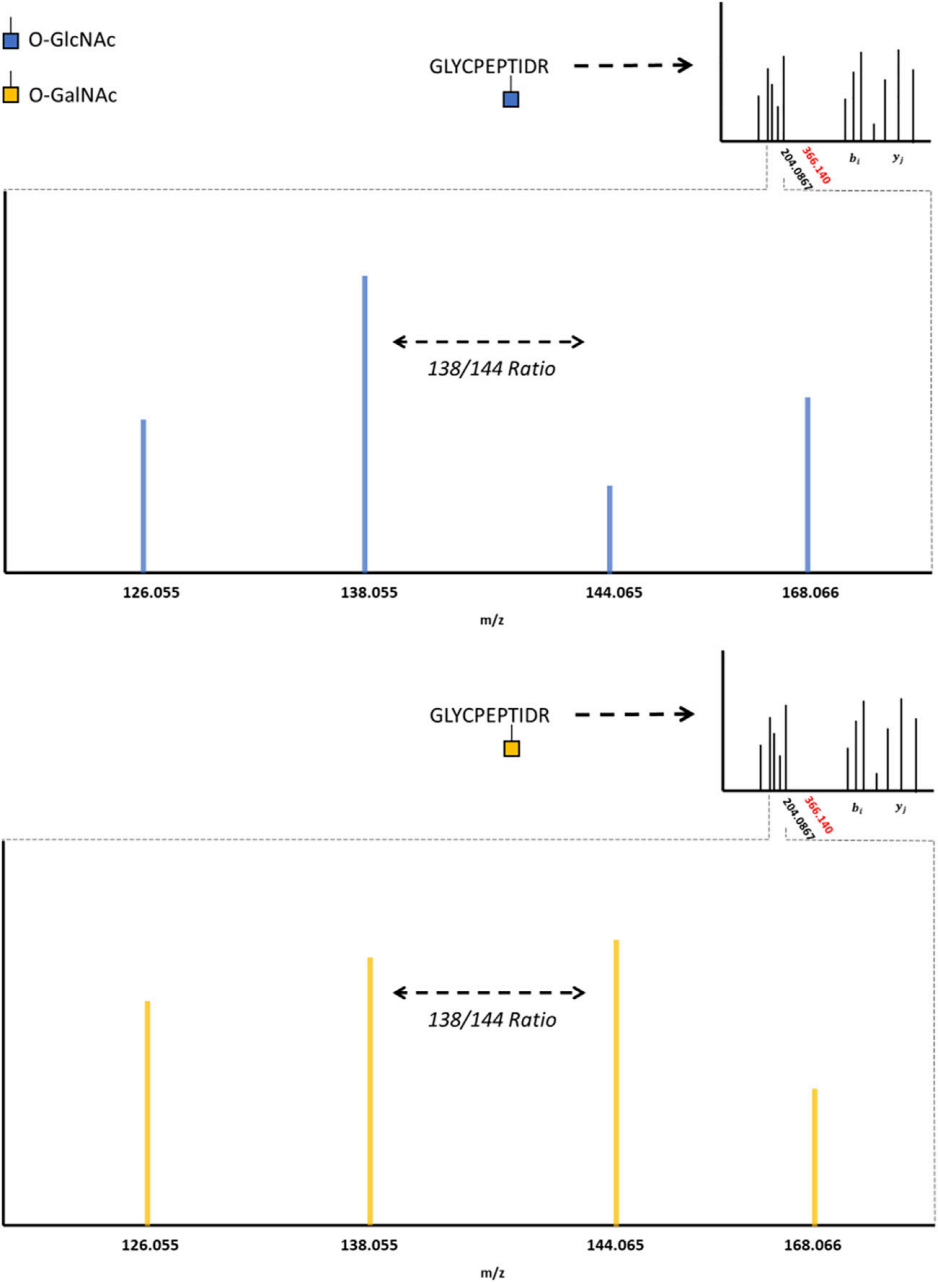
The O-GlcNAc Fingerprint HexNAc fragmentation patterns appear to be distinctive between O-GIcNAc (blue) and O-GalNAc (orange) stereoisomers. Upon collisional induced dissociation, the glycosidic bond between serine/threonine and the HexNAc modification is liberated, producing a characteristic 204.0867 peak. The liberated HexNAc ion can further fragment, producing the 168.066, 144.065, 138.055, and 126.055 m/z peaks. The relative ratio between these fragment peaks (such as the 138/144 ratio) are reported to be different between O-GIcNAc and O-GalNAc. The lack of a 366.140 peak, as indicated by the red marking on the spectrum inlet, indicates the absence of a HexHexNAc peak (the absence of a branched glycan). Importantly, these fragmentation patterns may vary between distinct HexNAc precursors and instrument settings; however, the trends distinguishing O-GIcNAc from O-GalNAc appear to be consistent (Halim et al., 2014; Burt et al., 2021; Pirro et al., 2021).

HexNAc modifications have distinctive fragment ions with HCD/CID methods. HexNAc collisional activation produces a characteristic HexNAc oxonium ion peak at 204.086 m/z (Carr et al., 1993; Huddleston et al., 1993; Chalkley and Burlingame, 2001). In beam-type instruments, the HexNAc oxonium ion can further fragment to produce a series of peaks, such as 186.076 m/z, 168.066 m/z, 144.065 m/z, 138.055 m/z, and 126.055 m/z (Peterman and Mulholland, 2006). Synthetic O-GlcNAc and O-GalNAc peptides were found to produce differential intensities of the 168.066, 144.065, 138.055, and 126.066 m/z peaks (Halim et al., 2014). These synthetic peptide standards suggested that O-GlcNAc oxonium ions favored the 168.066 and 138.055 m/z fragmentation pathways, where O-GalNAc oxonium ions favored the 144.065 and 126.055 m/z fragmentation pathways. This idea was later applied to complex proteome mixtures to resolve HexNAc modified peptides as either O-GlcNAc or O-GalNAc modified peptides using the 138.055/144.065 m/z ratio (Burt et al., 2021; Pirro et al., 2021)**.** As highlighted by Figure 2, HexNAc stereoisomer resolution for O-GlcNAc and O-GalNAc modified peptides is achieved when considering the relative abundance of HexNAc oxonium ion fragments for peptides sequenced with collisional activation methods. As GlcNAc can be incorporated into branched glycans, distinguishing a singular O-GlcNAc from a complex, branched N- or O-linked glycan is also important. Branched glycans typically produce a 366.140 m/z peak with HCD/CID, indicative of a HexHexNAc ion (Nilsson et al., 2010; Toghi Eshghi et al., 2016; Reily et al., 2019). As such, the absence of 366.140 m/z peak, the presence of 204.086 m/z peak, and a greater 138.055 m/z to 144.065 m/z ratio may provide a diagnostic O-GlcNAc “fingerprint” marker when applying HCD data acquisition methods.

### 1.4 Implications for Health and Disease

The global profiling of PTMs to investigate their function in health and disease is uniquely suited to mass spectrometry-based proteomics, as neither PTM localization nor stoichiometric abundance is genetically encoded. Bottom-up, DDA proteomic strategies localize and quantify the phosphoproteome (Humphrey et al., 2018; Mertins et al., 2018) (reviewed in (Riley and Coon, 2016)), the acetylproteome (Choudhary et al., 2009; Svinkina et al., 2015), and the ubiquitin modified proteome (Rose et al., 2016; Udeshi et al., 2020; Rivera et al., 2021), among other PTM spaces. The medical relevance of quantitative PTM mapping is highlighted when applied to patient-derived clinical samples. Examples include, but emphatically are not limited to, characterizing the proteomic landscape of cancer and identifying therapeutic vulnerabilities (Drake et al., 2016; Zagorac et al., 2018; Gillette et al., 2020; Hristova et al., 2020; Krug et al., 2020; Cao et al., 2021; Friedrich et al., 2021; Huang et al., 2021), mapping the physiological molecular transducers of exercise (Sanford et al., 2020), and characterizing differential PTM patterning in psychiatric and neurological disease (Martins-de-Souza et al., 2012; Sun et al., 2021). In contrast to phosphorylation, acetylation, and ubiquitination, global quantitative O-GlcNAc mapping in medically relevant contexts (i.e., patient-derived samples) is underdeveloped; however, the implication of aberrant O-GlcNAcylation contributing to disease states is well-documented.

#### 1.4.1 Cognitive Decline and Neurodegeneration

Relative to other organs, the brain is heavily O-GlcNAcylated (Cole and Hart, 2001; Wulff-Fuentes et al., 2021). Neuronal inducible OGT knockouts decreases the synaptic expression of the GluA2 and GluA3 subunits of the α-amino, 3 hydroxy-5 methyl-4 isoxazolepropionic acid receptor (AMPA) and decreases mature dendritic spine number (Lagerlöf et al., 2017). Neuronal OGT knockout mouse models elicit embryonic loss or early death in postnatal mice (O'Donnell et al., 2004), while constitutive loss in forebrain neurons shows neurodegeneration in adult mice (Wang et al., 2016). Moreover, the relatively intense localization of OGA to the adult human forebrain was observed via unique PET-OGA ligands (Paul et al., 2019; Lee J.-H. et al., 2020). Murine OGA brain-specific knockouts reduce pyramidal neurons in both the cortex and hippocampus and alter cortical layering while also inducing systemic metabolic changes (Olivier-Van Stichelen et al., 2017). Age-related loss of O-GlcNAcylation in neural stem cells alters a neuron-to-glia differentiation switch, contributing to a decline in adult hippocampal neurogenesis and neuronal dysfunction in older mice (Liu et al., 2012; White et al., 2020). Of note, two isoforms of OGT are highly expressed in the adult mammalian brain hippocampus, a full-length nucleocytoplasmic OGT isoform (ncOGT) and a short OGT isoform (sOGT). The expression of ncOGT selectively decreases during aging (Liu et al., 2012). The specificity of O-GlcNAcylation by the different OGT isoforms may play a role in regulating aging-associated and neurodegenerative phenotypes through targeting of different protein substrates. Subsequently, proteoform (a protein’s primary sequence in addition to its unique set of PTMs) identification and quantification of OGT/OGA in neural tissue may be a useful strategy. Top-down proteomics identifies proteins as compared to peptides thereof, allowing for experimental distinction between proteoforms (Kelleher et al., 1999; Smith et al., 2013). Top-down OGT/OGA proteoform characterization coupled to bottom-up O-GlcNAc site mapping would provide complementary information, the former on the mechanism driving differential O-GlcNAc patterning and the latter on the consequence of differential O-GlcNAc patterning.

Alzheimer’s disease (AD) is the leading cause of dementia. The hallmarks of AD include amyloid plaques and neurofibrillary tau tangles. Tau is a microtubule binding protein that regulates the stability of the neuronal cytoskeleton. In AD, tau is abnormally phosphorylated, and accumulates into toxic neurofibrillary tangles. (Kobayashi et al., 2003; Bejanin et al., 2017). Aberrant O-GlcNAcylation is observed in the AD brain (Griffith and Schmitz, 1995; Liu et al., 2004; Liu et al., 2009). Tau O-GlcNAcylation at Ser400 prevents phosphorylation at Ser 404 (Yuzwa et al., 2012). Conditional OGT loss in murine neural tissue increases hyperphosphorylation of tau (O'Donnell et al., 2004). Chronic pharmaceutical inhibition of OGA reduces pathological tau in the brain and cerebrospinal fluid of rTg4510 mice (transgenic mice exhibiting tauopathy) (Hastings et al., 2017). O-GlcNAcylation of tau stabilizes it from aggregation (Yuzwa et al., 2012). Enhanced neural O-GlcNAcylation may be a protective factor for tauopathy (Borghgraef et al., 2013), where hyperphosphorylated tau shows an inverse relationship with tau O-GlcNAcylation (Liu et al., 2009). As evident, promoting and/or monitoring tau O-GlcNAcylation may prove to be a useful strategy for tauopathies and dementia. Eli Lilly and Company has an OGA inhibitor (LY3372689) advancing to stage 2 clinical trial targeting tau O-GlcNAcylation for early symptomatic AD (ClinicalTrials.gov Identifier: NCT05063539). Alectos Therapeutics Inc. in partnership with Merck have completed phase 1 clinical trials for the OGA small molecule inhibitor MK-8719 (Sandhu et al., 2016). Global discovery approaches to quantitatively map the O-GlcNAc modified proteome in the context of neurodegenerative disease will be critical to not only characterize tau, but also to investigate other unknown consequences of perturbed O-GlcNAcylation. The relevance of the latter is underscored by the introduction of OGA inhibition as a pharmaceutical strategy.

#### 1.4.2 Cancer

Aberrant O-GlcNAcylation is implicated in numerous cancer types and cancer associated biological processes (Shi et al., 2010; Ma et al., 2013; Trinca et al., 2018; Jiang et al., 2019) (reviewed in (Slawson and Hart, 2011)). Global quantitative site mapping of O-GlcNAcylation in native tissue for tumor types reporting aberrant O-GlcNAcylation is a logical next step. Similar to other PTM spaces, mapping O-GlcNAcylation across different cancer types may provide both biomarker diagnostics and signaling mechanisms for therapeutic intervention. Moreover, native peptide O-GlcNAc enrichment methods that can be coupled to serial-PTM enrichment strategies could provide insight on signaling mechanisms between PTM spaces (i.e., “cross-talk”).

Beyond identification and abundance, important questions remain on the functional impact of O-GlcNAcylation on target proteins. One cancer related phenomenon influenced by O-GlcNAcylation is the Warburg effect (Shtraizent et al., 2017; Wang et al., 2017; Nie et al., 2020). The Warburg effect is a hallmark of cancer cells. It is defined by excessive glycolysis with an increased glucose uptake rate and preferential lactate production in the presence of oxygen (reviewed in (Liberti and Locasale, 2016)). The increased amount of glucose consumed is used as a carbon source for anabolic processes, supporting uncontrolled cellular proliferation. Glycolytic enzymes phosphoglycerate kinase 1 (PGK1) and pyruvate kinase M2 (PKM2) regulate this effect. These enzymes are also post-translationally modified by O-GlcNAc (Wang et al., 2017; Nie et al., 2020). Specifically, PGK1 is O-GlcNAcylated at threonine 255 (T255) leading to increased interaction with translocase of outer mitochondrial membrane 20 (TOM20) and translocation to the mitochondria. Mitochondrial PGK1 interacts with the pyruvate dehydrogenase (PDH) complex to decrease oxidative phosphorylation, promoting a Warburg phenotype (Nie et al., 2020).

To understand protein-protein interactions mediated by PGK1 T255 O-GlcNAcylation, O-GlcNDAz mediated photo-crosslinking followed by mass spectrometry could identify novel PGK1 mitochondrial interactors. GlcNDAz is an analog of GlcNAc in which the N-acyl position of GlcNAc is replaced with a photo-crosslinker. In cells expressing a mutant form (F383G) of UDP-GlcNAc pyrophosphorylase, a cell-permeable diazirine-modified form of GlcNAc-1-phosphate (GlcNDAz-1-P) is converted to UDP-GlcNDAz. OGT can transfer GlcNDAz from UDP-GlcNDAz to produce O-GlcNDAz modified PGK1, while not dramatically affecting the normal O-GlcNAcylation process. Cells are subsequently irradiated using UV light to activate the diazirine crosslinker, leading to covalent linkage between O-GlcNDAz modified PGK1 and neighboring molecules. The GlcNDAz modified substrate (PGK1) would then be immunoprecipitated, with a subsequent LC-MS/MS follow-up to determine interacting partners (Yu et al., 2012; Wu et al., 2022). This strategy could be applied to any OGT substrate/O-GlcNAc site; however, it is limited to *in vitro* applications.

## 2 Discussion

The capability to confidently map the O-GlcNAc modified proteome at the site-specific level has accelerated due to key advancements. O-GlcNAc enrichment strategies are numerous, poising their own advantages and disadvantages. ETD data acquisition strategies are landmark techniques for HexNAc site localization. In conjunction with product-triggered methods, both HCD and ETD fragmentation methods are leveraged to acquire relevant information of putative HexNAc modified precursors. HCD fragmentation may provide a “O-GlcNAc fingerprint” pattern through the dissociation of the HexNAc oxonium ion. This stereoisomer resolution will be critical for investigations that probe O-GlcNAcylation beyond cytoplasmic and nuclear proteins. HCD-pdt-ETD can leverage this information, providing TMT quantitation and HexNAc stereoisomer information in the HCD scan and HexNAc site localization in the ETD scan. Together, advancements in enrichment and data acquisition may provide the proper platform to investigate the role of this perplexing PTM in health and disease.
